# The Psychometric Properties of the Stress and Anxiety to Viral Epidemics-6 Items: A Test in the U.S. General Population

**DOI:** 10.3389/fpsyt.2021.746244

**Published:** 2021-10-06

**Authors:** Sangha Lee, Jihoon Lee, Soyoung Yoo, Sooyeon Suh, Seockhoon Chung, Sherman A. Lee

**Affiliations:** ^1^Department of Psychiatry, Ajou University Medical Center, Ajou University School of Medicine, Suwon, South Korea; ^2^Department of Psychiatry, Asan Medical Center, University of Ulsan College of Medicine, Seoul, South Korea; ^3^Department of Convergence Medicine, University of Ulsan College of Medicine, Seoul, South Korea; ^4^Department of Psychology, Sungshin Women's University, Seoul, South Korea; ^5^Department of Psychology, Christopher Newport University, Newport News, VA, United States

**Keywords:** COVID-19, stress, anxiety, scale, psychometry

## Abstract

**Objectives:** Many individuals around the world are suffering from psychological distress due to the COVID-19 outbreak. The aim of this study is to explore the validity and reliability of the English version of Stress and Anxiety to Viral Epidemics-6 (SAVE-6), which measures the anxiety response of the general population to the viral epidemic.

**Methods:** A cross-sectional web-based study with self-reporting measures was conducted. A total of 314 United States residents were recruited via online platform in exchange for payment. The participants were asked to an anonymous questionnaire, collecting information on demographics, psychiatric history, SAVE-6, Patient Health Questionnaire-4 (PHQ-4), and the Coronavirus Anxiety Scale.

**Results:** The result from confirmatory factor analysis (CFA) demonstrated that a single-factor model [$\chi_{\left(9\right)}^{2}$ = 11.53, *p* = 0.24] yielded excellent fit for all of indices [χ^2^/df ratio = 1.28; CFI = 1.00; TLI = 1.00; SRMR = 0.02; RMSEA = 0.03 (0.00, 0.07; 90% CI)] and yielded strong internal consistency reliability (Cronbach's α = 0.88). The results from multigroup CFAs showed that there were no gender differences [$\Delta \chi_{\left(6\right)}^{2}$ = 3.20, *p* = 0.78, ns] and no race differences [$\Delta \chi_{\left(6\right)}^{2}=$3.60, *p* = 0.73, ns] between the models, along with excellent model fits.

**Conclusions:** The results of this study support the reliability and validity of SAVE-6 with strong psychometric properties for the English version of the U.S. population.

## Introduction

A novel coronavirus disease 2019 (COVID-19) is a new infectious disease that occurred in Wuhan City, Hubei Province, China in December 2019. After the first outbreak, the COVID-19 has rapidly spread to neighboring countries, and in March of the following year, the World Health Organization (WHO) declared a pandemic, the highest level of warning of transmission, meaning the stage of a global pandemic. According to the World Health Organization, globally, as of September 9, 2021, there have been 221,648,869 confirmed cases of COVID-19, including 4,582,338 deaths, affecting 212 countries and territories. Especially in the United States of America, 41,300,407 confirmed cases of COVID-19 with 670,458 deaths have been reported ^1^.

The spread of COVID-19 has posed a great threat across social systems such as healthcare, public security, and the economy. Such rapid social change has had a profound effect individual mental health. The public has been exposed to constant fear and anxiety due to daily corona-related news (1). The fear of COVID-19 appears to be due to uncertainty about how much the current epidemic will deteriorate (2). According to a survey of Americans (3), 62% of respondents were more worried about COVID-19 than seasonal flu.

In addition, the public has experienced social isolation due to social distancing, working from home, and school closure, which can lead to various psychological problems such as personal stress, anxiety, depression, fear, anger, loneliness, frustration (4). Previous research has shown that people in quarantine suffered various psychological issues such as stress, fear, and depression (5). From the pandemics in the past, we have learned that there were more people affected by mental health than those affected by infections (6) and that mental health effects may be more lasting than the epidemic itself (7).

In response to these psychological crises brought about by COVID-19, researchers have developed measures to assess stress, anxiety, and fear specific to the pandemic. For example, Taylor et al. (8) developed a 36-item COVID Stress Scales (CSS) to measure a series of fears related to COVID-19. It demonstrated good validity and reliability in five factors, but the sample was limited to the US and Canadian populations and some items are believed to reflect sociocultural contexts (e.g., xenophobia toward Asians, insufficient supply in grocery stores). Another measure called the Fear of COVID-19 Scale (FCV-19S), proposed by Ahorsu et al., was designed to assess an individual's fear of COVID-19 with only seven items (9). Although FCV-19S is shorter (10), it contains items that focus primarily on physical reactions and appears to be limited to COVID-19 (e.g. “My hands become clammy when I think about coronavirus-19,” “My heart races or palpitations when I think about getting coronavirus-19”).

We originally developed Stress and Anxiety to Viral Epidemics-9 items (SAVE-9) scale, a nine-item scale to assess stress and anxiety of healthcare workers in response to the viral epidemic (11). It has the advantage of being a compact psychological scale that can be used in many various pandemic situations, and it was validated in various languages including Russian (12), Italian (13), Japanese (14), Turkish (15), and German (16). In a previous study, the SAVE-9 scale was divided into two factors; factor I- anxiety about viral epidemics (namely, SAVE-6), and factor II-work-related stress associated with viral epidemics. Although SAVE-9 is a well-established scale for measuring stress associated with viral epidemics, it is necessary to check the validity of the six-item item targeting the general public as it is for a specific occupational group. This scale is expected to be useful not only in the stress caused by COVID-19 but also in other pandemic situations that may occur in the future. We explored whether the SAVE-6 is useful for evaluating the anxiety related to the viral epidemic among the general population in Korea (17), and we found that it is a valid and reliable scale that may be used in the general population in Korea, Lebanon (18), and special population in Korea including cancer patients (19) and medical students (20). In this study, we aimed to assess the reliability and convergent validity of the English version of the SAVE-6 scale among the U.S. population.

## Materials and Methods

### Participants and Procedure

The data was collected via the online survey on December 11, 2020, from 314 adults residing in the United States, were used in this IRB approved study. The participants were recruited via Amazon MTurk in exchange for payment ($0.25) and were eligible if they provided consent and furnished complete information. Most of the participants (*Mage* = 39.53) were white (78.8%), female (52.2%), never diagnosed with COVID-19 (66.2%), knew someone who died of COVID-19 (61.5%), and plan on getting vaccinated for COVID-19 (78.8%) when they become available (see Table 1).

**Table 1:** Sample characteristics.

**Characteristics**	**Statistics**
**Gender**	
Male	150 (47.8%)
Female	164 (52.2%)
**Race**	
White	247 (78.7%)
Black	28 (8.9%)
Asian	20 (6.4%)
Hispanic	16 (5.1%)
Other	3 (1.0%)
**COVID-19 diagnosis**	
Yes	106 (33.8%)
No	208 (66.2%)
**Knowledge of someone who died of COVID-19**	
Yes	193 (61.5%)
No	121 (38.5%)
**Plans on getting vaccinated for COVID-19**	
Yes	247 (78.7%)
No	67 (21.3%)
**Age**	*M =* 39.53; *SD =* 11.46 (19–65)
**Symptoms ratings**	
Depression	*M =* 2.94*; SD =* 1.82 (0–6)
Generalized anxiety	*M =* 2.92*; SD =* 1.76 (0–6)
Suicidal ideation	*M =* 1.35*; SD =* 1.33 (0–4)
Substance use	*M =* 1.60*; SD =* 1.42 (0–4)
Coronaphobia	*M =* 7.87*; SD =* 5.91 (0–19)
Viral anxiety	*M =* 12.36*; SD =* 5.73 (0–24)

### Measures

To get composite scores, item ratings within a measure were combined together. Higher composite scores imply that a condition is more prevalent.

#### Basic Information

Participants were asked to report their age, gender, race, COVID-19 diagnosis, whether or not they knew someone who died of COVID-19, and whether or not they plan on getting vaccinated for COVID-19 when they are available.

#### Psychological Distress and Substance Use

Clinical depression and generalized anxiety were rated using the Patient Health Questionnaire-4 (PHQ-4) (21). Participants rated each items how frequently, within the past 2 weeks (0 = not at all to 3 = nearly every day), they experienced symptoms of depression (e.g., “feeling down, depressed, or hopeless.”) with two items (α = 0.78) and generalized anxiety (e.g., “feeling nervous, anxious, or on edge.”) (α = 0.74). Passive suicidal ideation was measured with the single item, “I wished I was already dead so I did not have to deal with the coronavirus.” While substance use coping was measured with the single item, “I used alcohol or other drugs to help me get through the fear and/or anxiety caused by the coronavirus.” Participants indicated how frequently, within the past 2 weeks (0 = not at all to 4 = nearly every day), they experienced suicidal thoughts and used alcohol or drugs to cope with coronavirus related fear and anxiety.

#### Coronaphobia

Clinical symptoms of anxiety that are tied to coronavirus related thoughts or information were measured using the Coronavirus Anxiety Scale (CAS) (22). Participants indicated how frequently, within the past 2 weeks (0 = not at all to 4 = nearly every day), they experienced symptoms of coronaphobia (e.g., “I felt dizzy, lightheaded, or faint, when I read or listened to news about the coronavirus.”) with five items (α = 0.93).

#### Viral Anxiety

General anxiety responses to the viral pandemic were measured using the Stress and Anxiety to Viral Epidemics-6 (SAVE-6) (17). Participants indicated their level of agreement (0 = never to 4 = always) with pandemic-related anxiety questions (e.g., “Are you afraid the virus outbreak will continue indefinitely?”) using six items (α = 0.88). See Table 2 for item properties.

**Table 2:** Results of exploratory factor analysis (EFA) of the SAVE-6 using principal component analysis with Oblimin rotation (*n* = 314).

**Item**	**Factor 1**
SAVE-6 item 2	0.795
SAVE-6 item 4	0.780
SAVE-6 item 3	0.743
SAVE-6 item 1	0.737
SAVE-6 item 5	0.717
SAVE-6 item 6	0.714
Eigenvalue	3.797
% of Variance	63.283
Cumulative variance	63.283

### Statistical Approach

A series of statistical analyses were used to examine the psychometric properties of the SAVE-6, a measure of viral anxiety. SAVE-6 total score differences in gender (men vs. women), race (whites vs. non-whites), COVID-19 diagnosis (yes vs. no), knowledge of someone who died of COVID-19 (yes vs. no), and plans on getting vaccinated for COVID-19 (yes vs. no), were examined using independent samples *t*-tests. SAVE-6 total score correlations with age and distress-related constructs (e.g., suicidal ideation) were examined using Pearson's product-moment correlations. Factor analysis was performed in two steps. In the first step, exploratory factor analysis (EFA) was conducted to determine using principal component analysis with Oblimin rotation to determine loadings of items and their dimensions. In the second step, a bootstrap (2,000 samples) maximum likelihood confirmatory factor analysis (CFA) was modeled on the six items of the SAVE-6 to examine the instrument's factorial validity for a unidimensional structure. Multigroup CFAs were run to determine if the SAVE-6 is measuring viral anxiety in the same way for men and women, as well as whites and non-whites. Satisfactory model fit for a CFA model was defined by a chi-square/df value <2, a standardized root-mean-square residual (SRMR) value ≤ 0.05, root-mean-square-error of approximation (RMSEA) value ≤ 0.10, and comparative fit index (CFI) and Tucker Lewis Index (TLI) values ≥ 0.90 (23, 24). Measurement invariance was defined by both adequate model fit statistics and a non-significant value (*p* ≥ 0.05) on a chi-square difference test. All of the statistical analyses were calculated using SPSS version 26.0, except for the confirmatory factor analyses (CFA), which were run using AMOS version 25.0.

## Results

### Descriptive Statistics, Group Comparisons, and Correlations

The descriptive statistics reveal that the majority of the sample were highly distressed during the COVID-19 pandemic. Specifically, 62.4% experienced clinical levels of depression (≥3) [Kroenke et al. (21)], 64.0% experienced clinical levels of generalized anxiety (≥3) (21), 51.6% experienced coronaphobia (≥9) (22), and 38.9% experienced high viral anxiety (≥15) (17). In addition, 58.6% had suicidal ideation and 65.3% coped with their fear and anxiety over the coronavirus using drugs or alcohol. Most of the participants plan on getting vaccinated for COVID-19 in the future (78.7%) and knew someone who died of COVID-19 (61.5%).

Viral anxiety was significantly greater among those with a COVID-19 diagnosis [*t*_(260.04)_ = 7.34, *p* < 0.001], those who knew someone who died of COVID-19 [*t*_(312)_ = 9.35, *p* < 0.001], and those who plan on getting the vaccine for COVID-19 [*t*_(94.81)_ = 4.78, *p* < 0.001]. Demographically, viral anxiety was slightly associated with age (*r* = 0.12), but not gender [*t*_(312)_ = 0.23, *p* = 0.82, ns] and race [*t*_(91.23)_ = 0.04, *p* = 0.97, ns]. In terms of distress-related constructs, viral anxiety was strongly associated with substance use coping (*r* = 0.61) and suicidal ideation (*r* = 0.59). As expected, and in support of the SAVE-6's construct validity, viral anxiety was shown to be associated with COVID-19 related experiences (i.e., COVID-19 diagnosis, knowledge of someone who died of the disease, and plans to get vaccinated) and distress-related constructs (e.g., suicidal ideation).

### Initial Exploratory Factor Analysis

**Figure 1:**
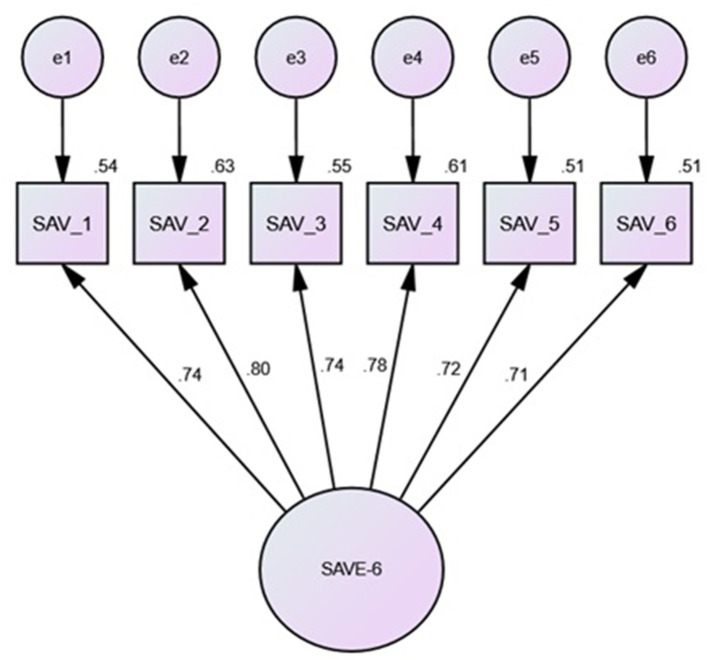
Confirmatory Factor Analysis. Note. Model based on bootstrap Maximum Likelihood (ML) estimations (2000 samples). All of the standardized coefficients are significant at the .05 level. SAV_1 = continuation fear; SAV_2 = health fear; SAV_3 = infection worry; SAV_4 = physical sensitivities; SAV_5 = avoidance worry; SAV_6 = transmission worry.

Table 2 and Figure 1 show the results of exploratory factor analysis of the SAVE-6 using principal component analysis with Oblimin rotation (*n* = 314). The analysis revealed one factors with an Eigenvalue > 1, explaining 63.3% of total variance. All included variables loaded highly on the factor.

### Confirmatory Factor Analyses

The SAVE-6 items were found to be acceptable for factor analysis after a preliminary examination of the data (25). Specifically, the data did not exhibit issues pertaining to sample size, missing data, non-normality, multicollinearity, or singularity. The correlation matrices were also shown to be factorable (Bartlett's test of sphericity = *p* < 0.001; Kaiser-Meyer-Olkin test = 0.90).

A CFA was used to see if the SAVE-6's six anxiety components could be combined into a unidimensional construct. The results demonstrated that a single-factor model [$\chi_{\left(9\right)}^{2}$ = 11.53, *p* = 0.24] yielded excellent fit for all of indices [χ^2^/df ratio = 1.28; CFI = 1.00; TLI = 1.00; SRMR = 0.02; RMSEA = 0.03 (0.00, 0.07; 90% CI)] and yielded strong internal consistency reliability (Cronbach's α = 0.88). Thus, these results support the factorial validity of the SAVE-6 measure (Table 3).

**Table 3:** Item properties of the SAVE-6.

	**Items**	**Response scale**					**Descriptive**		**Item metrics**		
		**0**	**1**	**2**	**3**	**4**	**M**	**SD**	**ITC**	***R* ^**2**^**	**CID**
1	Are you afraid the virus outbreak will continue indefinitely?	8.6%	15.9%	35.4%	25.8%	14.3%	2.21	1.14	0.69	0.48	0.86
2	Are you afraid your health will worsen because of the virus?	11.8%	19.7%	31.2%	29.3%	8.0%	2.02	1.13	0.74	0.55	0.86
3	Are you worried that you might get infected?	8.6%	18.8%	36.9%	20.7%	15.0%	2.15	1.15	0.69	0.50	0.86
4	Are you more sensitive toward minor physical symptoms than usual?	13.7%	20.7%	25.2%	27.4%	13.1%	2.05	1.25	0.72	0.53	0.86
5	Are you worried that others might avoid you even after the infection risk has been minimized?	21.0%	19.4%	24.5%	25.2%	9.9%	1.83	1.29	0.67	0.46	0.87
6	Do you worry your family or friends may become infected because of you?	14.6%	15.9%	29.9%	24.8%	14.6%	2.09	1.26	0.67	0.45	0.87

*Cronbach's Alpha is 0.88 for total SAVE-6 measure; # Item Number; 0, never; 1, rarely; 2, sometimes; 3, often; 4, always; M, Mean; SD, Standard Deviation; ITC, Corrected Item-Total Correlation; R^2^, Squared Multiple Correlation; CID, Cronbach's Alpha if item is deleted*.

Then, multiple sets of CFAs were run to check if SAVE-6's viral anxiety structure is measured in the same way on the demographic variables of gender (male vs. female) and race (white vs. non-white). The results show that there is no gender difference, which is evidenced by an excellent fit of the model. [$\chi_{\left(18\right)}^{2}$ = 23.10, *p* = 0.19] for all of the indices [χ^2^/df ratio = 1.28; CFI = 0.99; TLI = 0.99; SRMR = 0.03; RMSEA = 0.03 (0.00, 0.06; 90% CI)] and a non-significant increase in χ^2^ value [$\Delta \chi_{\left(6\right)}^{2}$ = 3.20, *p* = 0.78, ns] between the models. The results also demonstrated no race differences, which were evidenced by excellent model fit [$\chi_{\left(18\right)}^{2}$ = 25.87, *p* = 0.10] for all of the indices [χ^2^/df ratio = 1.44; CFI = 0.99; TLI = 0.98; SRMR = 0.02; RMSEA = 0.04 (0.00, 0.07; 90% CI)] and a non-significant increase in χ^2^ value [$\Delta \chi_{\left(6\right)}^{2}$ = 3.60, *p* = 0.73, ns] between the models. Thus, these results demonstrate measurement invariance by showing that the SAVE-6 measures viral anxiety the same way across gender and race groups.

### Evidence Based on Relations to Other Variables

The SAVE-6 scale score was significantly correlated with PHQ-4 anxiety subscale (*r* = 0.67, *p* < 0.001), PHQ-4 depression subscale (*r* = 0.64, *p* < 0.001), or CAS scale (*r* = 0.74, *p* < 0.001).

## Discussion

The aim of the current study was to assess the psychometric properties of SAVE-6, a newly developed scale designed to evaluate the anxiety level associated to COVID-19 pandemic. The psychometric properties of the SAVE-6 were assessed in a representative sample of 314 adults who were between 19 and 65 years of age in the USA. The current study confirmed and extended previous reports of reliability and validity (17).

The result indicated that the internal consistency of SAVE-6 (Cronbach Alpha=.88) is excellent and adequate for CFA (Bartlett's test of sphericity = *p* < 0.001; Kaiser-Meyer-Olkin test = 0.90). The SAVE-6 score significantly correlated with depression and GAD scores, as well as another anxiety scale specific to COVID-19 (CAS), indicating good convergent validity. Previous studies have reported that people who have been diagnosed with COVID-19 or who knew someone who died of COVID-19 were more likely to meet the anxiety and depression criteria (26). Corona-related structures and viral anxiety in this study appeared to be higher in those with corona-related experiences, which seems to be consistent with these existing studies. Perceived stress associated with the coronavirus is a strong predictor of higher dysfunction and can predict symptoms of depression and anxiety disorders. In addition, we were able to confirm measurement invariance in all groups using multiple-group CFA. As shown in the results, gender and race did not seem to affect the response pattern of SAVE-6. Therefore, it can be concluded that the SAVE-6 is a reliable measure that assesses psychological issues associated with a viral epidemic across cultures.

The SAVE-6 is a rating scale which can measure the anxiety response specifically to the viral epidemic. It includes items asking anxiety symptoms such as “Are you afraid the virus outbreak will continue indefinitely?,” “Are you afraid your health will worsen because of the virus?,” or “Are you worried that you might get infected?” We believe the anxiety symptoms measured with this scale might be viral anxiety and not anxiety stemming from other factors. Although several measures have recently been published for COVID-19-related fears and anxiety, SAVE-6 differs from other measures in several ways. The COVID-19 Stress Scale developed by Taylor et al. include social contexts such as socioeconomic consequences of COVID, xenophobia, and compulsive checking (8). Other rating scales have been proposed to assess the symptoms of anxiety and associated physiological arousal (the Coronavirus Anxiety Scale) (22), nervousness, muscle tensions, or behaviors of avoidance (the COVID-19 Anxiety Questionnaire) (27), or avoidance, checking, and worried behaviors (the COVID-19 Anxiety Syndrome Scale) (28). Even other scales such as FCV-19S (9) or Coronavirus Pandemic Anxiety Scale (CPAS-11) (29) are similar to SAVE-6 in that it is evaluating the primary fear/anxiety of coronavirus, SAVE-6 differs in that it responds not only to the COVID-19 but also to other virus pandemics. With the possibility of another unpredictable pandemic that may occur in the future, we believe the scale will have additional utility in the future. We have explored the validity of the SAVE-6 among the general population in Korea (17), and it has reported that the scale has reliable psychometric properties. The SAVE-6 has been validated in other languages (18). In particular, the English version of SAVE-6 is expected to be highly utilized in a number of English-speaking countries.

This study has some limitations. First, all data was collected via online self-report surveys, which may have potential bias or errors. Further research involving various methods of assessment, such as face-to-face interviews or focus group interviews may enrich the analysis. Second, at the time of our survey, other measures were being reviewed and yet to be published, so the concurrent validity with them could not be confirmed. If the concurrent validity with the aforementioned scales can be reviewed later, it will help to increase the validity of SAVE-6. Third, some demographic characteristics such as education level, employment status, medications, history of psychiatric illness, and income level were not available. Since they were not included in covariates, some possible confounding factors may remain. Despite the limitation, the results of this study support the reliability and validity of SAVE-6 with strong psychometric properties for the English version of the U.S. population.

## Data Availability Statement

The raw data supporting the conclusions of this article will be made available by the authors, without undue reservation.

## Ethics Statement

The studies involving human participants were reviewed and approved by the Institutional Review Board of Christopher Newport University. Written informed consent for participation was not required for this study in accordance with the national legislation and the institutional requirements.

## Author Contributions

SC, SS, and SAL: conceptualization and writing—review and editing. SAL: methodology, formal analysis, data curation, and visualization. JL, SY, and SL investigation. SAL and SL: writing—original draft preparation. SS: supervision. SC: project administration and funding acquisition. All authors have read and agreed to the published version of the manuscript.

## Funding

This work was supported by the Framework of International Cooperation Program managed by the National Research Foundation of Korea (FY2020K2A9A1A01094956).

## Conflict of Interest

The authors declare that the research was conducted in the absence of any commercial or financial relationships that could be construed as a potential conflict of interest.

## Publisher's Note

All claims expressed in this article are solely those of the authors and do not necessarily represent those of their affiliated organizations, or those of the publisher, the editors and the reviewers. Any product that may be evaluated in this article, or claim that may be made by its manufacturer, is not guaranteed or endorsed by the publisher.

## Footnotes

^1^Available online at: https://covid19.who.int/ (accessed September 9, 2021).
